# First Case of Small Bowel Sarcomatoid Carcinoma Found by Video Capsule Endoscopy

**DOI:** 10.4021/gr414w

**Published:** 2012-03-20

**Authors:** Mohit Mittal, Dhavan A. Parikh, Heidi Jess, Surinder K. Mann

**Affiliations:** a Internal Medicine, UC Davis Medical Center, Sacramento, CA, United States; b Gastroenterology, UC Davis Medical Center, Sacramento, CA, United States; c Pathology, UC Davis Medical Center, Sacramento, CA, United States

**Keywords:** Video capsule endoscopy, Small bowel tumor, Sarcomatoid carcinoma

## Abstract

Sarcomatoid carcinoma of the small bowel is extremely rare. We report the first case of sarcomatoid carcinoma identified by video capsule endoscopy in a patient referred for obscure gastrointestinal bleeding. Computed tomography and small bowel follow through failed to identify the tumor. The tumor was visualized initially on video capsule endoscopy examination and a 6 x 3 cm polypoid, fungating mass with irregular borders was retrieved on surgical resection. Microscopic examination showed sheets of pleomorphic spindled to epitheliod cells staining positive for cytokeritin and vimentin, indicative of sarcomatoid carcinoma. Forty-one months after surgical resection the patient continued to be free of metastatic disease.

## Introduction

Video capsule endoscopy (VCE) is a relatively safe and widely utilized endoscopic imaging method of the small bowel (SB), and is a notable advancement in the endoscopic identification of small bowel tumors (SBTs). Prior to VCE the prevalence of SBTs was thought to account for 1-3% among all gastrointestinal malignancies [1]. Since the introduction of VCE in 2001, many studies have indicated the true prevalence of SBTs to be substantially higher [2]. Primary carcinoma of the SB is rare with an incidence of 0.5 - 0.8 per 100,000 population per year [3, 4]. Sarcomatoid Carcinoma (SCA) is an extremely rare and malignant subtype of adenocarcinoma of the SB, of which only 24 cases have been reported in the English literature to date [5-9]. Here we present the first case of a jejunal SCA occurring in a middle-aged Caucasian man identified by VCE.

## Case Report

**Figure 1:**
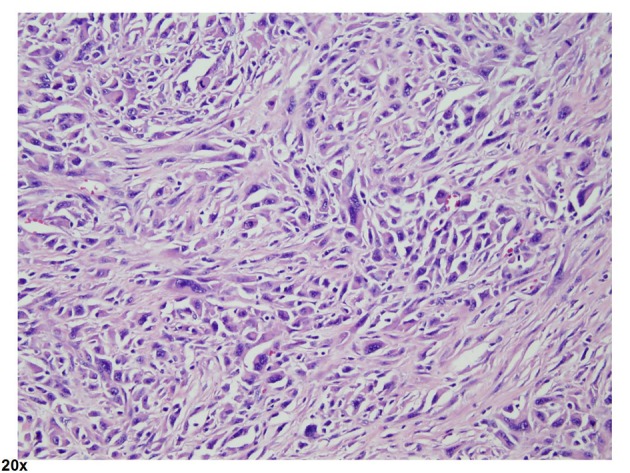
Hematoxylin and eosin (H and E) stain. Pleomorphic spindle cells with moderate amount of eosinophilic cytoplasm, irregular nuclear membranes, vesicular chromatin and prominent nucleoli. Numerous mitotic figures are noted. Objective magnification x 20

**Figure 2:**
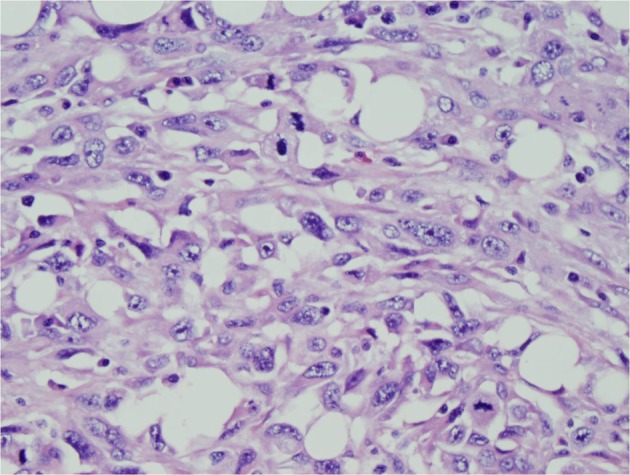
Hematoxylin and eosin (H and E) stain. Pleomorphic spindled to epitheliod cells with moderate amount of eosinophilic cytoplasm, vesicular chromatin and prominent nucleoli.  Numerous mitotic figures are noted. Scattered plasma cells are seen in the background.  Objective magnification x 40.

**Figure 3:**
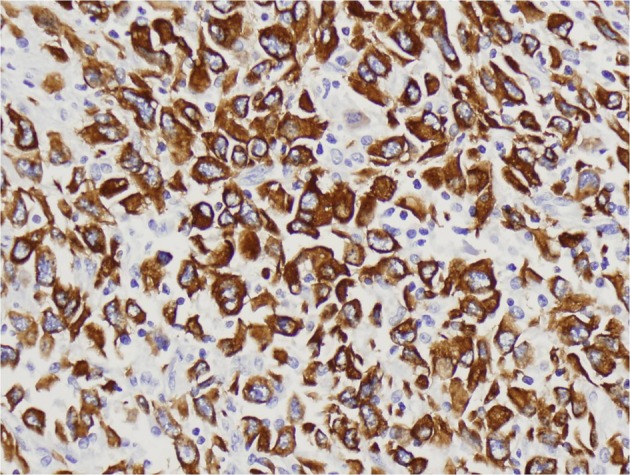
Positive staining for CAM 5.2 immunohistochemical stain. Objective magnification x 50.

**Figure 4:**
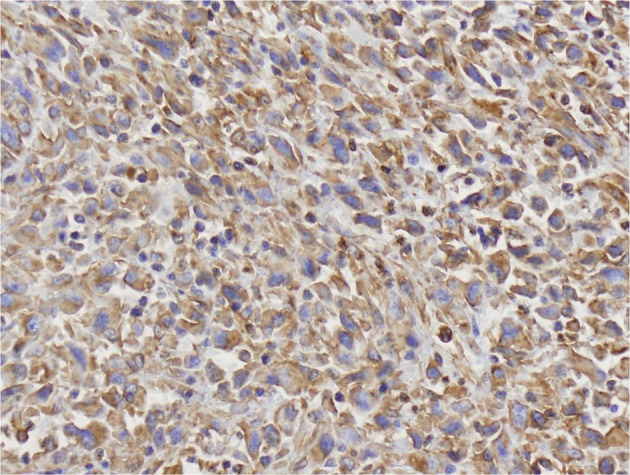
Positive staining for Vimentin immunohistochemical stain. Objective magnification x 50.

A 69 year-old man with COPD and an active 80 pack year smoking history presented to his primary care physician with fatigue, dizziness and intermittent melena for 1 month. He was found to have iron deficiency anemia with a hemoglobin of 8.3g/dL, iron saturation of 4%, and a ferritin of 24 ng/mL. Examination by esophagogastroduodenoscopy (EGD) and colonoscopy showed no abnormal findings. Small bowel follow through was also negative for any pathology. The patient was then referred for VCE which showed a large chronic ulcer suspicious for malignancy. An abdominal computed tomography (CT) scan was done to further characterize the lesion but did not identify the primary lesion or any sites of metastasis. The patient then underwent surgical resection of the jejunal ulcer. A 6 x 3 cm polypoid, fungating tumor with irregular borders was retrieved without evidence of mesenteric adenopathy or hepatic metastases. Microscopically the tumor was seen infiltrating the normal enteric mucosa with sheets of pleomorphic spindle cells. The cells had a moderate increase in nuclear to cytoplasmic ratios, vesicular chromatin and prominent nucleoli (Fig. 1, 2). Tumor cells showed immunoreactivity to anti-cytokeritin marker and Vimentin (Fig. 3, 4) consistent with SCA. There was no evidence of lymphovascular or perineural invasion and the surrounding lymph nodes were free of metastasis. Follow-up forty-one months after diagnosis showed the patient to be free of metastatic disease.

## Discussion

Tumors of the SB are rare. The SB makes up 75% of the length and 90% of the absorptive area of the GI tract [10], yet far fewer cancers originate in the SB. Diagnosis of SBTs is also difficult as many patients are asymptomatic or present in a non-specific manner. As such, malignant SBTs often present late and almost 50% have metastasized at the time of presentation [11]. Prognosis of malignant SBTs is generally poor with curative surgical resection as the mainstay of treatment in cases where SBTs are identified prior to metastases. The advent of VCE has led to earlier diagnosis and therapeutic intervention of SBTs [12]. Video capsule endoscopy has also been shown to diagnose SBTs earlier in patients with Lynch Syndrome [13]. Identifying tumors early while at a resectable stage is of paramount importance in increasing survival.

Small bowel tumors have four major histologic subtypes: adenocarcinoma, neuroendocrine tumor, gastrointestinal stromal tumor, and lymphoma. Sarcomatoid carcinoma of the SB is an extremely rare and malignant form of adenocarcinoma. Sarcomatoid carcinomas have been reported in diverse organ systems including respiratory, digestive, salivary, thyroid, breast and skin with the esophagus being the most common site of gastrointestinal SCAs [14-16]. On histopathology, SCA can have either a monophasic pattern consisting of predominantly a mesenchymal-like component, or a biphasic pattern composed of both epitheloid and mesenchymal components [15]. Immunohistological staining of SCA tumors is usually positive for epithelial markers cytokeritin and vimentin. Other epithelial makers can be identified as well including epithelial membrane antigen, carcinoembryonic antigen, and Leu-M1 [14, 16, 17].

According to Reid-Nicholson et al the prognosis for those diagnosed with SCA is much worse compared to other tumors of the SB. Of the 20 cases reviewed, patients had large and advanced tumors at the time of diagnosis. Seventy percent of patients died between 2 months to 3 years after diagnosis with 79% having metastatic or recurrent disease at the time of death. No definite risk factors were identified. Surgical resection was the mainstay of treatment, as patients showed poor response to chemotherapy and radiation treatment alone [5].

Here we report the first case of primary jejunal SCA identified by VCE. In our case both small bowel follow through and CT were negative for any pathology, suggesting that VCE was able to identify the lesion prior to the development of radiographic findings. The general higher sensitivity of VCE is supported by Saurin et al who showed that VCE is superior to CT enteroclysis in identifying SBTs in patients with Lynch Syndrome [13]. Further, Hara et al suggested the superiority of VCE compared to small bowel follow through and CT in identifying all small bowel pathology [18]. Pathological examination confirmed that the tumor was of early stage with no nodal dissemination. Our patient has had the longest survival of any known patient with SCA of the SB consisting currently of forty-one months without any evidence of metastatic disease.

The prognosis for SCA of the SB is usually poor with metastasis often at the time of presentation. Video capsule endoscopy has the potential to identify tumors at a resectable stage. This case displays the importance of early referral for VCE for evaluation of obscure gastrointestinal bleeding as it can lead to early diagnosis, resection, and improved prognosis of this malignant neoplasm.
